# Glycobiology of the Epithelial to Mesenchymal Transition

**DOI:** 10.3390/biomedicines9070770

**Published:** 2021-07-02

**Authors:** Michela Pucci, Nadia Malagolini, Fabio Dall’Olio

**Affiliations:** Department of Experimental, Diagnostic and Specialty Medicine (DIMES), General Pathology Building, University of Bologna, Via San Giacomo 14, 40126 Bologna, Italy; michela.pucci3@unibo.it (M.P.); nadia.malagolini@unibo.it (N.M.)

**Keywords:** glycosylation, glycosyltransferases, galectins, carbohydrate antigens, glycolipids

## Abstract

Glycosylation consists in the covalent, enzyme mediated, attachment of sugar chains to proteins and lipids. A large proportion of membrane and secreted proteins are indeed glycoproteins, while glycolipids are fundamental component of cell membranes. The biosynthesis of sugar chains is mediated by glycosyltransferases, whose level of expression represents a major factor of regulation of the glycosylation process. In cancer, glycosylation undergoes profound changes, which often contribute to invasion and metastasis. Epithelial to mesenchymal transition (EMT) is a key step in metastasis formation and is intimately associated with glycosylation changes. Numerous carbohydrate structures undergo up- or down-regulation during EMT and often regulate the process. In this review, we will discuss the relationship with EMT of the *N*-glycans, of the different types of *O*-glycans, including the classical mucin-type, *O*-GlcNAc, *O*-linked fucose, *O*-linked mannose and of glycolipids. Finally, we will discuss the role in EMT of galectins, a major class of mammalian galactoside-binding lectins. While the expression of specific carbohydrate structures can be used as a marker of EMT and of the propensity to migrate, the manipulation of the glycosylation machinery offers new perspectives for cancer treatment through inhibition of EMT.

## 1. Introduction

Glycosylation consists in the enzymatically mediated addition of single sugars or sugar chains to proteins or lipids, giving rise to the formation of glycoproteins or glycolipids, respectively. A significant proportion of the mammalian genomes encodes molecules involved in the addition (glycosyltransferases) removal (glycosidases), recognition (lectins) of sugars, as well as in other glycosylation-related functions. The majority of cell membrane or secreted proteins, including growth factor receptors [1], cell adhesion molecules [2] and antibodies [3] are indeed glycoproteins [4]. Glycosylation serves many biological functions [5], often acting as a “fine tuner” of the cellular and molecular interactions [6]. Cancer-associated glycosylation changes are responsible for several aspects of malignancy, including proliferation, invasion and metastatization [7,8,9,10,11]. Epithelial to mesenchymal transition (EMT) is a crucial step in the physiological processes of embryogenesis and wound healing and, in cancer, of metastasis formation. In EMT, epithelial cells lose their intercellular contacts, mainly because of E-cadherin down-regulation, change morphology, acquiring a fibroblastoid shape, and increase motility and migration. This process is triggered by a variety of stimuli, in particular by transforming growth factor-β (TGF-β) which, through SMAD signaling [12], leads to profound changes in gene expression. EMT is a reversible process, which can be reverted by the mesenchymal to epithelial transition (MET). EMT is also associated with glycosylation changes [13]. In this review, we will discuss how the different classes of carbohydrate chains are modulated by EMT and how glycosylation can regulate the EMT process.

## 2. Essentials of Glycosylation

**Figure 1:**
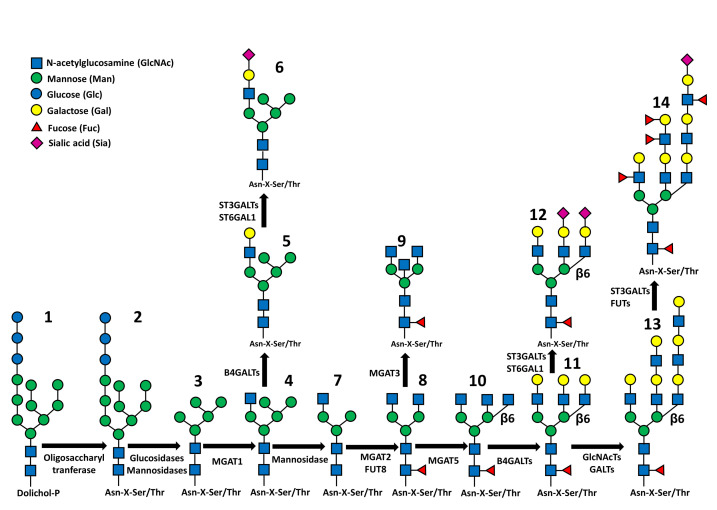
Synthetic representation of *N*-glycan biosynthesis. Structures 1 and 2: the Glc_3_Man_9_GlcNAc_2_ structure attached to dolichol phosphate (1) or to polypeptide (2). Structure 3: Man_5_GlcNAc_2_ structure product of the trimming of 4 Man and 3 Glc residues. Structure 4: addition of the first outer β1,2-linked GlcNAc. Structures 5 and 6: hybrid type structures in which 5 Man residues coexist with a fully processed branch. Structure 7: trimming of the last 2 Man residues. Structure 8: addition of the second outer β1,2-linked GlcNAc and of “core-linked” fucose. Structure 9: addition of the “bisecting” β1,4-linked GlcNAc. Structure 10: addition of the β1,6-linked GlcNAc. Structure 11: addition of β1,4-linked Gals. Structure 12: addition of some Sia residues either α2,3 or α2,6-linked. Structure 13: Elongation of the branches by sequential action of GlcNAcTs and GalTs. Structure 14: addition of sialic acids and external fucose residues. The fucosylated structure present on the left branch could be either the Le^a^ or the Le^x^ structure, depending on the Fuc linkage to GlcNAc (α1,4 for Le^a^; α1,3 for Le^x^). The fucosylated structure present on the internal branch could be either the Le^b^ or the Le^y^ structure, depending on the Fuc linkage to GlcNAc as above. The β1,6-linked branch on the right is terminated by a sialylated Lewis^a^ or Lewis^x^ antigen.

The two main types of glycoprotein glycosylation include the *N*-glycosylation, in which glycans are attached to the amidic nitrogen of asparagine through their terminal GlcNAc residue, and the *O*-glycosylation, in which sugars are attached to the hydroxyl group of serine or threonine, usually through their GalNAc terminal residues. These two types of glycans are synthesized through completely different mechanisms. In fact, while the biosynthesis of *O*-linked chains proceeds through the stepwise addition of the single monosaccharides to the oligosaccharide chains of glycoproteins en route along the exocytic pathway [14], the *N*-glycosylation is based on more complex mechanisms [15,16]. The first step of *N*-glycosylation takes place in the rough endoplasmic reticulum (RER) and consists in the building of an oligosaccharide comprised of 2 GlcNAc, 9 Man and 3 Glc residues on the dolichol-phosphate lipid carrier (Figure 1, structure 1), through the sequential action of specific glycosyltransferases. Then, this “high mannose” oligosaccharide is transferred en bloc by the oligosaccharyltransferase complex on a Asn-X-Ser/Thr consensus motif of a nascent polypetide chain (structure 2). The presence of this motif, in which X represents any amino-acid except proline, is a necessary but not sufficient condition for oligosaccharyltransferase action. After transfer to protein, the high mannose oligosaccharide undergoes the sequential trimming of the 3 Glc residues and of 4 Man residues (structure 3). At this stage, the glycoprotein moves to the Golgi apparatus, where the successive biosynthetic steps take place. On a terminal Man of this Man_5_GlcNAc_2_ structure a GlcNAc residue is added (structure 4). This is the first step of the biosynthesis of an outer branch (or *antennae*) which leads to the conversion of the high mannose glycan into a “complex type” glycan. After GlcNAc addition, 2 additional Man residues are trimmed, leaving the structure GlcNAc_1_Man_3_GlcNAc_2_ (structure 7), which is the substrate for other GlcNAc transferases adding the first sugars of additional branches (structures 8 and 10) or the bisecting GlcNAc (structure 9). The structure is completed by galactose, sialic acid and fucose residues (structures 11, 12). While sialic acid is always in terminal position of the branches, fucose can be attached both to the innermost GlcNAc residue (core fucosylation, structure 8) or to branches often elongated by repeated GlcNAc-Gal units, forming Lewis antigens (structures 13, 14). In some cases, the first GlcNAc attached to Man is elongated with Gal and Sia without trimming of the last two mannose, giving rise to “hybrid type” glycans (structures 5, 6). *N*-linked structures exhibit a remarkable level of variability, which is strictly related to their biological functions. 

## 3. General Relationship of Glycosylation with EMT

In this section, we will discuss of the EMT/glycosylation relationship in general, regardless of specific carbohydrate structures. There are several examples of how EMT alters the glycosylation process and vice versa. TGF-β-induced EMT caused in a non-malignant bladder cell line an increased proportion of hybrid-type and fucosylated complex type glycans [17] and an increase of hybrid type glycans and a reduction of *N*-linked branching, fucosylation and bisecting in a normal mouse mammary cell line [18]. Modulation of the genes *ALG9* (encoding a α1,2 Man transferase), *MGAT3* and *MGAT4B* was consistent with these structural changes [18]. Increased expression of hybrid and complex-type glycans was observed also in human normal mammary epithelial cells induced to shift towards a mesenchymal appearance by soluble factors from malignant cells [19]. In A459 cells, EMT resulted in increased glucose uptake, which was largely shifted to the hexosamine biosynthesis pathway (HBP). This led to increased availability of the donor substrate UDP-GlcNAc, which was used by GlcNAc transferases in multiple glycosyltransferase reactions. As a consequence, several glycan structures of the cell surface, often associated with cancer, were altered [20]. In a study on the impact of EMT on glycogene expression in pancreatic cancer cells treated with TGF-β and in panels of pancreatic cell lines exhibiting either epithelial or mesenchymal morphology, it was found that the mesenchymal condition was associated with increased proteoglycan biosynthesis [21] and that some glycosyltransferases displayed differential expression in mesenchymal-like cells [21]. In hepatocellular carcinoma cells, EMT induced by HGF reduced the expression of MGAT3 but increased that of MGAT5, FUT8 and B3GALT5 [22]. Sialylation is a key feature of glycosylation. Using a metabolic labeling of sialylated glycans, a general decrease of sialylation, in particular on β4 integrins, was observed in TGF-β-induced EMT [23]. The level of expression of different sialyltransferases usually increased, with the remarkable exception of ST6GALNAC1 which decreased [23]. Altogether, these data point to the accumulation of hybrid-type glycans as an EMT-associated feature observed in several, but not all the systems studied. 

There also examples of how glycosylation affects the EMT process. One regards the inhibitory immune membrane receptor PD1 and its ligand PD-L1. The ectopic expression of PD-L1 by cancers is responsible for inhibition of T lymphocyte activity and consequent escape from immunosurveillance. EMT-activated β-catenin stimulated the transcription of the STT3 components of the oligosaccharyltransferase complex. The consequent increased *N*-glycosylation of PD-L1 resulted in its stabilization and enhancement of its biological activity [24]. Another example was provided by SNAIL which regulates transcription of *CRB3* (Crumbs3a), a small transmembrane protein required to generate the apical membrane and tight junctions. In SNAIL-induced EMT, CRB3 glycosylation was altered, resulting in its reduced membrane half-life and an increase of the depolarizing process of EMT [25].

## 4. *N*-Glycosylation

The degree of branching and the core fucosylation are crucial features of *N*-glycans involved in EMT regulation. 

### 4.1. Branching

A crucial step in the regulation of *N*-glycan branching is represented by the mutually exclusive action of GlcNAc transferases MGAT5 and MGAT3 (Figure 1). The first catalyzes the addition of the “β1,6-branching” and is associated with malignancy, while the second synthesizes the “bisecting GlcNAc” and is associated with reduced malignancy, mainly by preventing the action of the first. Here, we review the evidences supporting the role of the two enzymes and their cognate carbohydrate structures. The promoting role of MGAT5/β1,6-branching has been observed in different systems. In keratinocytes from MGAT5-overexpressing mice, migration, wound healing and EMT were increased [26]. In hepatocarcinoma, MGAT5 enhanced the interaction of the extracellular matrix metalloproteinase inducer (EMMPRIN/Basigin/CD147) with integrin β1 and promoted EMT and metastasis [27]. The drug fluvastatin slows down the growth of metastatic breast cancer cells by reducing branching through MGAT5 down-regulation [28]. Cells of the retinal pigment epithelium (RPE) induced to EMT displayed increased β1,6-branching of *N*-linked chains, which resulted in increased galectin-3 binding [29]. MGAT5 inhibition by the drug PST3.1a reduced invasiveness, proliferation and EMT of glioma cancer stem cells [30]. MGAT5 reduced radiosensitivity and increased malignancy in lung cancer cells [31]. The effect of MGAT5 was evident also on the promotion of physiological EMT in a mouse model of liver fibrosis in which KO of MGAT5 reduced the process [32]. 

The role of MGAT3/bisecting GlcNAc in EMT inhibition was supported by the following observations. In hepatocarcinoma, TGF-β-induced EMT resulted in down-regulation of MGAT3 through activation of Smad3 and Erk1/2 [33]. In a non-tumorigenic mouse mammary cell line, TGF-β treatment induced EMT and a concomitant down-regulation of *Mgat3* through promoter methylation [34]. MGAT3 inhibited EMT in breast cancer cells even when EMT was induced by hypoxia [35]. 

Several studies showed the relationship between the two enzymes and their related structures. In the human pre-neoplastic MCF10A cell line, TGF-β-induced EMT resulted in decreased MGAT3 expression and slightly increased MGAT5 expression. In turn, forced MGAT3 expression reduced EMT through the expression of the bisecting GlcNAc on cell adhesion molecules, such as E-cadherin and β1 integrins, demonstrating a causative role of this modification [36]. In the same cell line, EMT-like alterations associated with decreased MGAT3 and increased MGAT5 expression could be induced also by growth in sparse conditions [37]. In lung cancer cells, TGF-β induced EMT through non-muscle myosin II-A/ JNK/ P38/PI3K axis, resulting in a concomitant up-regulation of MGAT5 and down-regulation of MGAT3 [38]. In hepatocellular carcinoma cells, HGF-induced EMT was associated with decreased expression of MGAT3 and increased MGAT5 expression [22]. In ovarian and triple negative breast cancer cells, treatment with the DNA methyltransferase inhibitor 5-AZA-2-deoxycytidine activated MGAT5 transcription, resulting in increased migration and EMT [39], highlighting the potential side effects of epigenetic therapeutics in cancer treatment. Although EMT is usually promoted by MGAT5 and inhibited by MGAT3, sometimes the opposite is true. It has been proposed that this could depend on a differential effect of the two enzymes when cells are in a transition state between EMT and MET [40]. Besides the two above discussed enzymes, recent data support an EMT-promoting role also for MGAT1 [41]. In one of the studies cited above [28], fluvastatin was shown to inhibit also MGAT1.

### 4.2. Core Fucosylation 

The phenotype of the *Fut8* KO mice, which displayed attenuation of TGF-β1 receptor signaling resulting in increased activity of matrix matalloproteinases (MMPs) and emphysematous lesions in the lung [42], demonstrated the importance of core fucosylation for TGF-β1 signaling. Consequently, in a variety of systems core fucosylation was found to be a potent inducer of EMT. FUT8 was increased in EMT-induced by HGF in hepatocellular carcinoma cells [22] and in non-small cell lung cancer (NSCLC). In the latter, FUT8 activated the β-catenin/LEF-1 transcription factor [43], while it was inhibited by miR-198-5p overexpression, resulting in EMT inhibition [44]. FUT8 promoted EMT through core fucosylation of TGF-β receptor in both breast cancer cells [45] and in rat peritoneal mesothelial cells [46]. In the former, FUT8 could be activated by the opioid analgesic fentanyl and promoted EMT and malignancy through the β-catenin pathway [47]. The physiological EMT induced in renal tubular cells through TGF-β1 receptors can be blocked by inhibition of FUT8 [48]. 

## 5. *O*-Glycosylation

**Figure 2:**
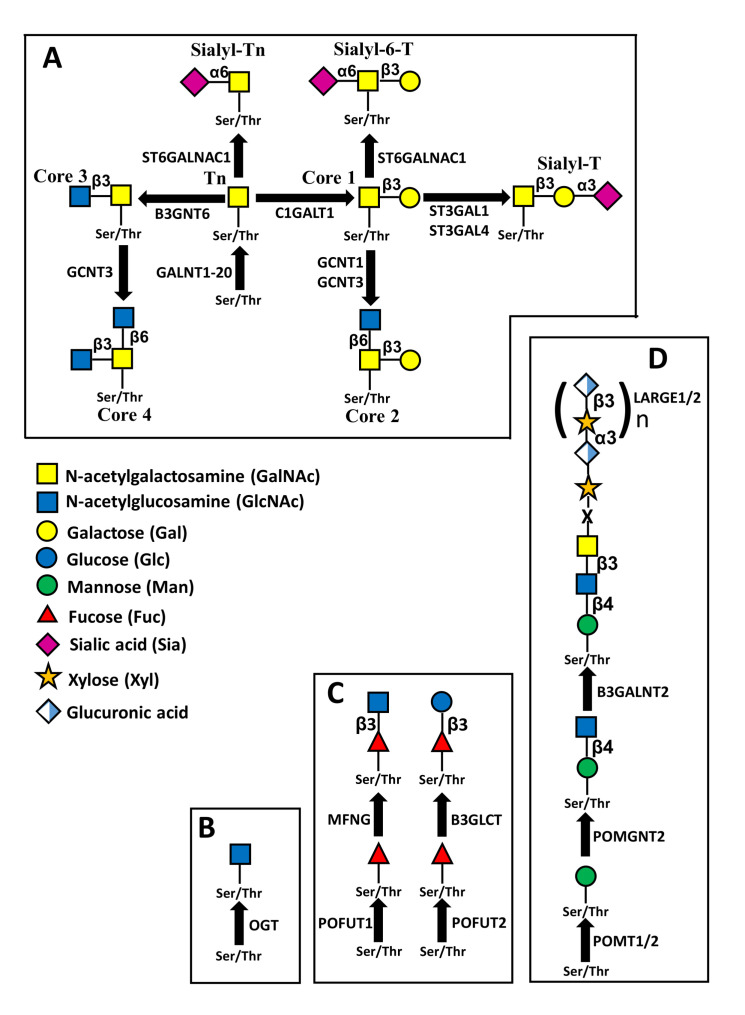
Structures of different types of *O*-linked chains. (**A**) structure and biosynthesis of mucin-type *O*-linked chains. Four “core” structures are shown, which can be elongated by other sugars. (**B**) Addition of *O*-linked GlcNAc, mediated by *O*-GlcNAc transferase. Removal of the sugar is catalyzed by O-GlcNAc ase (not shown). (**C**) *O*-fucosylation. The *O*-linked fucose mounted on EGF repeats by POFUT1 can be elongated by β3GlcNAc through manic fringe (MFNG) and also by β4Gal and α6Sia (not shown). The *O*-linked fucose mounted on TSRs repeats by POFUT2 can be elongated by β3Glc (**D**) *O*-mannosylation. The structure shown is referred to as M3 and can be elongated by the repeated units of xylose and glucuronic acid.

In the classical mucin-type *O*-glycosylation, the sugar linked to peptide is GalNAc (Figure 2A). Through the cooperative and competitive activity of different glycosyltransferases, different carbohydrate structures are generated. Besides well defined carbohydrate structures, which are often cancer associated antigens, such as sialyl-Tn and sialyl-T, in Figure 2A are reported four “core” structures (core1-core4) which can be elongated through the action of various glycosyltransferases, forming more complex glycans. Other sugars which can be *O*-linked to peptide are GlcNAc (Figure 2B), Fuc (Figure 2C) and Man (Figure 2D).

### 5.1. Mucin Type O-Glycosylation

The addition of GalNAc to peptide, first step of mucin-type *O*-glycan biosynthesis, can be mediated by 20 different GalNAc transferases (GALNT1 to GALNT20), with subtle differences in substrate specificity [49]. Accumulation of truncated *O*-linked chains (Tn antigen) has been shown to promote EMT in different systems and a number of GALNT enzymes display association with EMT. In fact, in human prostate epithelial cells, TGF-β-induced EMT was associated with increased expression of *O*-glycosylated oncofetal fibronectin and of GALNT activity, while in pancreatic cancer cells undergoing TGF-β-induced EMT, GALNT2 and GALNT10 were increased [21]. A causal role of *O*-glycosylation in EMT was indicated by the finding that TGF-β-induced EMT was inhibited by KO of GALNT3 and GALNT6 [50]. Consistently, GALNT3 expression was inhibited by ZEB2, which is a transcriptional repressor and EMT inhibitor [51]. The positive relationship between GALNT enzymes and EMT was confirmed by GALNT14 in a breast cancer cell line [52] and by GALNT5 in cholangiocarcinoma [53]. On the contrary, in pancreatic cancer cells with mesenchymal morphology, GALNT3 was found to be reduced, compared with cells with epithelial appearance [21]. Besides the overexpression of GALNTs, the accumulation of unprocessed GalNAc can be due also to the lack of expression of the β1,3 galactosyltransferase C1GALT1 (Figure 2A), which elaborates the core 1 structure. This enzyme requires the presence of the molecular chaperone COSMC for its activity. In pancreatic cancer cells, the KO of COSMC resulted in Tn accumulation, increased EMT and malignancy [54]. In ovarian cancer cells, EMT was stimulated also by the expression of sialyltransferase ST3GAL1, which synthesizes sialyl-T (Figure 2A) [55]. Yet, EMT is promoted also by core 2 and core 4 structures, as shown by the observation that in NSCLC, inhibition by miR-302b-3p of the GlcNAc transferase GCNT3, which mediates the biosynthesis of both structures, hindered cell growth and EMT [56]. On the other hand, core 3 structure promoted the opposite process: the mesenchymal to epithelial transition (MET) [57]. In fact, expression of core 3 synthase B3GNT6 led to decoration by core 3 structures of the membrane-tethered MUC1 N terminus, inhibiting its translocation to the nucleus and allowing p53 transcription [57].

### 5.2. O-GlcNAcylation 

Unlike canonical kinds of glycosylation, which modify extracellular and membrane molecules, the addition of *O*-GlcNAc modifies cytoplasmic and nuclear proteins [58]. The addition of *O*-linked GlcNAc is mediated by a single enzyme: *O*-GlcNAc transferase (OGT). *O*-GlcNAcylation is a dynamic process in that this single sugar can be detached and reversibly replaced by phosphate groups on Ser/Thr residues. *O*-GlcNAcylation is dependent on the availability of the donor substrate UDP-GlcNAc which, in turns, acts as a sensor of the glucose concentration through HBP. In A459 cells undergoing EMT, glucose is increasingly taken-up and largely shifted to HBP. The increased *O*-GlcNAcylation affects EMT [20]. When Ser 124 of the receptor for activated C kinase 1, product of the *RACK1* gene (formerly GNB2L1), was *O*-GlcNAcylated, the protein level of this receptor, which is a crucial mediator of EMT in gastric cancer metastasis, was dramatically reduced [59]. *O*-GlcNAcylation of EZH2, a transcriptional repressor involved in EMT, resulted in its stabilization and increased EMT and metastatic ability of CRC [60].

### 5.3. O-Fucosylation

Fucose can be directly *O*-linked to serine or threonine residues located within consensus sequences contained in EGF-like or thrombospondin type 1 repeats (TSRs) by protein *O*-fucosyltransferases 1 and 2 (POFUT1 and POFUT2), respectively (Figure 2C) [61]. NOTCH receptors are very rich of EGF repeats and are major substrates for POFUT1. In this case, *O*-linked fucose can be elongated by the addition of a β1,3 GlcNAc, catalyzed by Manic Fringe, product of the *MFNG* gene, and successively by Gal and Sia. In a group of poor prognosis breast cancers characterized by low claudin level, stemness and mesenchymal features, *MFNG* behaves such as an oncogene, promoting NOTCH signaling [62]. Trophoblast cells must undergo EMT to allow mouse embryo implantation. The process is promoted by the growth factor epiregulin which increased *O*-fucosylation mediated by POFUT1 of urokinase-type plasminogen activator [63]. On the other hand, *O*-fucosylation of TSRs is mediated by POFUT2 and can be elongated by β1,3 Glc, mediated by B3GLCT (Figure 2C). During mouse embryonic development, *O*-fucosylation of TSRs mediated by *Pofut2* is necessary to inhibit EMT, ensuring proper gastrulation [64]. 

### 5.4. O-Mannosylation

α-dystroglycan is a plasma membrane glycoprotein indirectly connecting the actin filaments of the cytoskeleton with the laminin of the extracellular matrix. The interaction of α-dystroglycan with laminin is mediated by its *O*-mannosyl glycans. The one depicted in Figure 2D is the most complex and is referred to as M3 structure (reviewed in [65,66]). The extended chain of these glycans by repeating units of a disaccharide formed by glucuronic acid and xylose is necessary for laminin binding. Both sugars of the repeating unit are added by the enzyme products of the genes *LARGE1* and *LARGE2*. In prostate cancer, EMT is induced by hypoglycosylation of α-dystroglycan, which is due to inhibition of *LARGE2*, by binding to its promoter of the trascriptional repressor ZEB1 and SNAIL [67]. 

## 6. Chain Elongation

Both, the *N*-linked chains and the mucin-type *O*-linked chains can be modified by extended linear or branched sugar chains. In this section, the contribution of these extended structures to EMT is reviewed. B3GALT5 is a key enzyme for the biosynthesis of type 1 lactosaminic chains and is dramatically down-regulated in colorectal cancer [68]. However, in other systems it is associated with malignancy and EMT. In fact, in hepatocarcinoma cells induced to EMT by HGF treatment, the expression of B3GALT5 was increased [22]. In breast cancer cells, B3GALT5 enhanced migration, invasion, mammosphere formation and EMT, through upregulation of β-catenin and ZEB1 [69]. On the other hand, B4GALT3, a key enzyme involved in the biosynthesis of type 2 lactosaminic chains, was increased in cervical cancer and associated with malignancy and EMT [70]. The linear type 2 lactosaminic structures (referred to as “i” structures) present in early infancy, become progressively more branched with growth, due to the increasing activity of a β1,6 GlcNAc transferase, encoded by the *GCNT2* gene, generating the “I” antigen. GCNT2 has been reported to promote EMT in at least three different systems. In breast cancer cells, TGF-β-induced EMT was inhibited upon GCNT2 inhibition [71]; in esophageal squamous cell carcinoma, overexpression of GCNT2 induced migration and invasion and EMT [72]; in colon cancer cells, EMT induced by EGF or bFGF was associated with up-regulation of GCNT2 through down-regulation of miR-199a/b-5p [73].

## 7. Chain Termination

In this section, we will discuss the structures which are often present at the end of sugar chains, namely sialylated and fucosylated structures.

### 7.1. Sialylation

Sialyltransferase ST6GAL1 is the main, if not the only, sialyltransferase able to add α2,6-linked sialic acid to Galβ4GlcNAc (type 2) chains, such as those usually present in *N*-linked chains. ST6GAL1 is overexpressed in colon cancer [74] and other malignancies [8]. According to several studies, ST6GAL1 promoted malignancy [75], although others reported the opposite [76,77]. A promoting effect of ST6GAL1 on EMT was suggested by some studies. In osteosarcoma cell models, KO of ST6GAL1 resulted in decreased malignancy and reduced expression of EMT-associated markers [78]; TGF-β-induced EMT of the mouse epithelial cell line GE11 induced ST6GAL1 up-regulation, while its down-regulation reduced EMT [79]; in pancreatic cancer cells, EGFR-mediated EMT was increased in cells highly expressing ST6GAL1 [80]. On the contrary, in triple-negative breast cancer cells, EMT was promoted by ST6GAL1 down-regulation by mir-214-3p [81].

Polysialic acid (PSA) is a linear polymer of α2,8-linked Sia residues which decorates, in a strictly stage-specific manner, a restricted set of cell surface glycoproteins, particularly in the central nervous system. Polysialylation of glycoproteins, in particular of the neural cell adhesion molecule (NCAM) strongly affects intercellular adhesion. Polysialylation of NCAM is mediated by two sialyltranferases: ST8SIA2 [82] and ST8SIA4 [83]. In mouse mammary cells, TGF-β-induced EMT resulted in increased PSA expression [84]. According to another study in the same mouse cellular model, TGF-β-induced EMT required the presence of the prion protein (PRNP). This protein is a normal component of the membranes of neural cells which, when aberrantly folded, is responsible for neurodegenerative diseases, such as Creutzfeldt-Jacob and “mad cow” diseases. PRNP induced the transcription of the ST8SIA2 gene and eventually the polysialylation of NCAM [85,86].

### 7.2. Fucosylation

**Figure 3:**
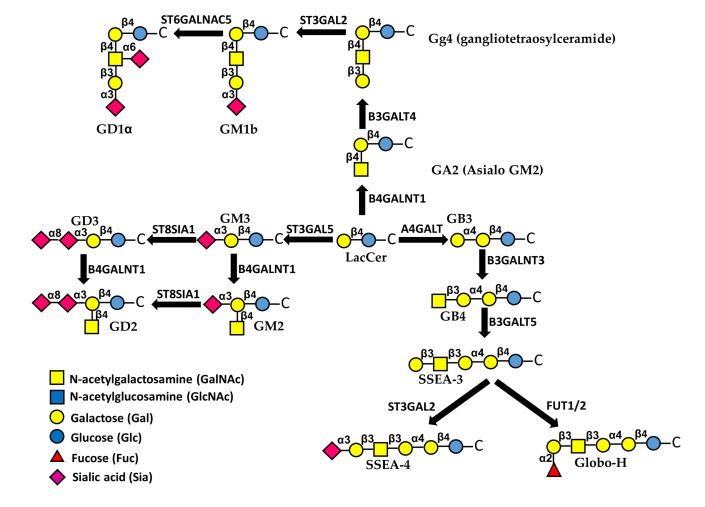
Biosynthesis and structure of major glycolipids. The first sugar attached to ceramide (C) is always Glc, followed by a β1,4-linked Gal. This basic structure can be elongated through different pathways. Sialylated glycolipids are referred to as gangliosides.

Terminal fucosylation includes the biosynthesis of the H antigen, of the Lewis^a^ (Le^a^), Lewis^b^ (Le^b^), Lewis^x^ (Le^x^), Lewis^y^ (Le^y^) and the sialylated counterparts of Le^a^ (sialyl Lewis^a^, sLe^a^) and of Le^x^ (sialyl Lewis^x^, sLe^x^). In colon cancer cells undergoing EGF/bFGF-induced EMT, FUT2, one of the two fucosyltransferases which synthesize the H antigen (Figure 3) was down-regulated, suggesting its EMT-preventing role [87]. By contrast, in lung adenocarcinoma, FUT2 supported TGF-β-induced EMT, in that its KO resulted in increased E-cadherin expression and decreased expression of EMT markers [88]. The involvement of FUT4, which mainly elaborates the Le^y^ antigen, in EMT was supported by some observations. Type 2 anti-inflammatory macrophages (M2) are frequently associated with cancers and thought to play a cancer-promoting activity. M2 macrophages have been shown to induce EMT in lung cancer cells through TGF-β release which, in turns, up-regulated FUT4 and Le^y^ antigen expression on ezrin receptor of cancer cells. Fucosylated ezrin enhanced its phosphorylation, promoting EMT [89]. An EMT-promoting activity of FUT4 was confirmed by studies in lung cancer cells [90] and in breast cancer [91]. FUT3 and FUT6 are among the fucosyltransferases able to synthesize the sLe^x^ antigen, while FUT3 is the only enzyme able to synthesize sLe^a^. Their reaction takes place after the addition of α2,3-linked sialic acid, catalyzed by various ST3GALs. Several lines of evidence suggest an EMT-promoting role of FUT3 in colon cancer cells. First, during EGF/bFGF-induced EMT, transcription of FUT3 (as well as that of ST3GAL1, −3, −4) and expression of their cognate sLe antigens, was increased [87]. Second, inhibition of FUT3/6, reduced fucosylation of TGF-β receptor and consequently reduced phosphorylation of downstream molecules and EMT [92,93]. Consistent results were obtained by FUT3 inhibition in pancreatic cancer cells [94]. On the other hand, FUT1 and FUT3, as well as Le^y^ antigen, were found to be highly expressed in various epithelial cells and reduced in EMT, suggesting an opposite effect [95]. 

## 8. Glycolipids

Glycosphingolipids are membrane glycoconjugates comprised of the hydrophobic ceramide portion embedded in the plasma membrane and a carbohydrate portion facing outside the cell [96]. Sialic acid containg glycolipids are referred to as gangliosides [97,98] (Figure 3). Glycolipids modulate many aspects of cell behavior through different mechanisms, including their interactions with growth factor receptors [1]. Specific glycosphingolipids are known to mediate EMT, sometimes in opposite directions [99,100]. In general, it appears that EMT is promoted by gangliosides but inhibited by neutral glycolipids. In fact, the neutral gangliotetraosylceramide (Gg4) and its biosynthetic enzyme B3GALT4 (Figure 3) were both down-regulated during EMT induced by hypoxia in normal murine mammary gland cells [101]. This was due to direct binding of the Smad3/4 complex to the B3GALT4 promoter [102]. Moreover, Gg4 was closely associated with E-cadherin and β-catenin and forced B3GALT4 expression inhibited EMT [101]. An anti-EMT activity was exerted also by globosides (Figure 3). In fact, cholera-toxin-induced EMT was allowed only upon depletion of A4GALT, the biosynthetic enzyme of globoside 3 (Gb3) [103]. Interestingly, a globoside-derived sialylated glycolipid, the stage-specific embryonic antigen-4 (SSEA-4), displayed an opposite behavior. In fact, it marked a subpopulation of chemotherapy-resistant breast cancer cells with mesenchymal features and the level of expression of its rate-limiting enzyme ST3GAL2 was associated with poor clinical outcome in breast and ovarian cancer patients treated with chemotherapy [104].

The EMT-promoting activity of gangliosides was supported by the observation that in human epithelial cells, TGF-β-induced EMT was mediated by an increase of ganglioside GM3 and its synthase ST3GAL5 [105] (Figure 3). Interestingly, ST3GAL5 is among the glycosyltransferases regulated by miRNAs of the miR-200 family, known to regulate EMT [106]. The addition to GM3 of a α2,8-linked sialic acid residue, catalyzed by sialyltransferase ST8SIA1 (GD3 synthase), and a β1,4-linked GalNAc residue, catalyzed by B4GALNT1 generates di-sialo gangliosides GD3 and GD2, respectively (Figure 3). GD2 was a marker of cancer stem cells and increased dramatically during EMT in transformed human mammary epithelial cells. Inhibition of ST8SIA1 resulted in a reduced number of cancer stem cells and reduced EMT [107]. Mechanistically, EMT regulates *ST8SIA1* by the binding to its promoter of the transcription factor FOXC2, a central downstream effector of several EMT pathways, and the activation of the c-Met pathway [108]. Altogether, these data support the notion that sialylation is a key factor in determining the pro- or anti-EMT activity of glycolipids. In MDCK cells stably expressing sialyltransferase ST6GALNAC5, which mediates the conversion of GM1b to GD1α (Figure 3) the *HGF* gene was up-regulated and signaling of its receptor MET was increased, resulting in EMT [109]. Consistently, ST6GALNAC5 was identified through a miRNA proxy approach as one of the key glycosyltransferases regulating EMT [106]. 

## 9. Galectins

Galectins are soluble galactose-binding lectins which mediate a variety of cellular functions, including cell growth, apoptosis, immune recognition and stemness [110]. Galectins, which are inherently di- or multivalent, can be localized extracellularly, on the cell membrane or intracellularly [111,112]. Owing to their multivalency, galectins can cross-link glycans on cell adhesion molecules and growth factor receptors, forming a lattice which stabilizes receptors on the cell membrane, modulating their signaling [1,113]. For this reason, some members of the galectin family play a role in EMT regulation. Galectins regulate EMT in cancer, as well as in other physio-pathological processes, mainly associated with fibrosis.

By binding to different membrane receptors, triggering different pathways, galectin-1 promoted EMT in a variety of systems. Through up-regulation of integrins, galectin-1 increased invasiveness and EMT of squamous carcinoma [114]. By stimulation of the toll-like receptor 4 (TLR4) with bacterial lipopolysaccharide (LPS), colon cancer cells release galectin-1 which induced lactate production and EMT [115]. On the other hand, in ovarian cancer cells, galectin-1 released in response to TLR4 induced EMT through the phosphatidylinositol 3-kinase (PI3K)/AKT pathway [116]. In gastric cancer cells overexpressing galectin-1, EMT was mediated through a sphingosine-1-phosphate receptor 1-dependent mechanism [117], resulting in increased malignancy [118]. In ovarian cancer cells, forced galectin-1 overexpression induced EMT through the MAPK JNK/p38 pathway [119]. Transfection experiments revealed that in hepatocarcinoma cells, galectin-1 induced EMT through the Wnt and PI3K/Akt pathways [120,121]. Galectin-1 produced by cancer cells or by normal cells of the microenvironment is a major regulator of the cancer-stroma crosstalk. In fact, galectin-1 produced by hepatocarcinoma cells controls the proliferation and migration of liver sinusoidal endothelial cells and their interaction with hepatocarcinoma cells [122]. Galectin-1 released by fibroblasts associated with gastric cancer, promote gastric cancer cells EMT, by binding to integrins and activating the hedgehog pathway [123,124]. Pancreatic stellate cells are myofibroblast-like cells that, through the expression of matrix molecules, influence the behavior of both normal and cancer pancreatic cells. Galectin-1 released by pancreatic stellate cells induced EMT of pancreatic ductal adenocarcinoma cells by activating the NF-kB pathway [125]. Two studies report an apparent conflicting role of galectin-3 in cancer. In fact, galectin-3 has been reported to support the progression through the Wnt pathway in oral squamous cell carcinoma [126] and to inhibit EMT and malignancy in breast cancer stem cells [127].

There are several examples of galectin-regulated EMT in non-neoplastic diseases. First, in a mouse model of age-related macular degeneration (AMD), galectin-1 was involved in neovascularization and subretinal fibrosis, which is the end-stage of AMD [128]. EMT of RPE cells is a crucial event in the onset of proliferative vitreoretinopathy (PVR), the most common reason for failure of retinal detachment surgery. As mentioned above, RPE cells undergoing EMT displayed increased galectin-3 binding because of increased β1,6-branching of *N*-linked chains [29] and inhibit attachment and spreading [129]. Idiopathic pulmonary fibrosis is a chronic condition characterized by the differentiation of epithelial cells and fibroblasts into matrix-secreting myofibroblasts. In mice KO for the *LGAL3* gene, encoding galectin-3, fibrosis and TGF-β-induced EMT were dramatically reduced [130]. In spontaneously hypertensive rats, galectin-3 prevents renal damage fibrosis and EMT [131]. In MDCK cells, galectin-8 promoted an oncogenic-like transformation through partial EMT, associated with higher proliferation and invasion [132].

## 10. Conclusions

In this review, we have discussed the relationship between glycosylation and EMT. We have shown that structures such as the β1,6-branching, core fucose and gangliosides promoted EMT in all the systems investigated, while other structures, including bisecting GlcNAc and neutral glycolipids, always inhibit EMT (Table 1). The possibility to target carbohydrate structures for cancer therapy has stemmed a variety of approaches, many of which are currently in clinical trials, as recently reviewed [133].

The glycan structures more frequently considered as potential therapeutic targets are among those involved in EMT and include short *O*-glycans, such as Tn, sTn, sT (Figure 2) and gangliosides (Figure 3). These structures have been targeted by cancer vaccines, monoclonal antibodies (Mabs) and CAR-T cells. The generation of vaccines targeting cancer-associated carbohydrates has to cope with their poor antigenicity, due to their nature of T-independent antigens and to the fact that they are self antigens. These problems have been circumvented by conjugation of sugar epitopes with heterologous proteins, forming conjugated vaccines. One of the first and the most famous was Theratope, which consisted in multiple sTn molecules linked to keyhole limpet haemocyanin (KLH). Unfortunately, Theratope was found to be ineffective on breast [134] and other cancers. A pentavalent vaccine formed by two glycolipids (Globo-H and GM2) and three short *O*-glycans (Tn, sTn and Core 1 structure) linked to KLM was found to be able to induce a serological response in ovarian cancer patients after a Phase I trial [135]. Treatment with the Mabs Dinutuximab and Racotumomab has been found to increase disease-free and overall survival of neuroblastoma [136] and NSCLC [137] patients. The first Mab targets ganglioside GD2, while the second Mab targets *N*-glycolyl-GM3. Targeting of GD2 in neuroblastoma patients was pursued also by the use of CAR-T cells, yielding a complete remission in one third of the patients [138]. Besides these successful stories, other attempts to cure cancer by targeting carbohydrate epitopes failed, while many other clinical trials are ongoing and expected to be completed in the next few years [133]. The future developments of the carbohydrate-targeted therapies are largely dependent on the success of these trials.

## Figures and Tables

**Table 1:** Summary of the effects of various carbohydrate structures on EMt.

Carbohydrate Chains	Structure(s)	Enzyme(s)	Evidences Pro-EMT	Evidences Anti-EMT
*N*-linked	β6-branching	MGAT5	[22,26,27,28,29,30,31,32,37,38,39]	
	Bisecting GlcNAc	MGAT3		[22,33,34,35,36,37,38]
	Core Fucose	FUT8	[22,43,44,46,47,48]	
*O*-linked mucin type	Tn	GALNT1-20; COSMC	[21,50,51,52,53,54]	[21]
	Sialyl-T	ST3GAL1	[55]	
	Core 2, Core 4	GCNT3	[56]	
	Core 3	B3GNT6		[57]
Other *O*-glycans	*O*-GlcNAc	OGT	[59,60]	
	*O*-Fuc	POFUT1,POFUT2, MFNG	[62,63]	[64]
	*O*-Man	LARGE1,LARGE2		[67]
Chain elongation	Type 1 chains	B3GALT5	[22,69]	
	Type 2 chains	B4GALT3	[70]	
	I antigen	GCNT2	[71,72,73]	
Sialylation	Sia6LacNAc	ST6GAL1	[78,79,80]	[81]
	Polysialylation	ST8SIA2	[84,85,86]	
Fucosylation	H-antigen	FUT1, FUT2	[88]	[87,95]
	Le^y^	FUT4	[89,90,91]	
	sLe^a^,sLe^x^	FUT3, FUT6	[87,92,93,94]	[95]
Neutral glycolipids	Gg4	B3GALT4		[101,102]
	Gb3	A4GALT		[101,103]
Gangliosides	SSEA-4	ST3GAL2	[104]	
	GM3	ST3GAL5	[105,106]	
	GD3, GD2	ST8SIA1	[107,108]	
	GD1α	ST6GALNA5	[106,109]	

## Abbreviations

AMD	age-related macular degeneration
bFGF	basic fibroblast growth factor
CAR-T cells	chimeric antigen receptor-T cells
EMT	epithelial to mesenchymal transition
EGF	epidermal growth factor
Gb3	Globoside 3
Gg4	gangliotetraosylceramide
HBP	hexosamine biosynthesis pathway
HGF	hepatocyte growth factor
KLH	keyhole limpet haemocyanin
Le^a^	Lewis^a^
Le^b^	Lewis^b^
Le^x^	Lewis^x^
Le^y^	Lewis^y^
LPS	lipopolysaccharide
Mab	monoclonal antibody
MET	mesenchymal to epithelial transition
MMP	matrix metalloproteinases
NCAM	neural cell adhesion molecule
NSCLC	non-small cells lung cancer
PD1	programmed death 1
PRNP	prion protein
PSA	polysialic acid
PVR	proliferative vitreoretinopathy
RER	rough endoplasmic reticulum
RPE	retinal pigment epithelium
sLe^a^	sialyl Lewis^a^
sLe^x^	sialyl Lewis^x^
SSEA-4	stage-specific embryonic antigen-4
TGF-β	transforming growth factor-β
TLR4	Toll-like receptor 4
TSRs	Thrombospondin type 1 repeat
